# Anthracycline cardiotoxicity: current methods of diagnosis and possible role of ^18^F-FDG PET/CT as a new biomarker

**DOI:** 10.1186/s40959-023-00161-6

**Published:** 2023-03-27

**Authors:** Mônica M. C. Becker, Gustavo F. A. Arruda, Diego R. F. Berenguer, Roberto O. Buril, Daniela Cardinale, Simone C. S. Brandão

**Affiliations:** 1 Postgraduate Program in Surgery, Federal University of Pernambuco, Recife, State of Pernambuco Brazil; 2 Recife Medical School, Federal University of Pernambuco, Recife, State of Pernambuco Brazil; 3 Postgraduate Program in Translational Health, Federal University of Pernambuco, Recife, State of Pernambuco Brazil; 4 Cardioncology Unit, European Institute of Oncology, I.R.C.C.S., Milan, Italy; 5 Nuclear Medicine Department, Hospital das Clínicas, Federal University of Pernambuco, 1st floor, 1235 Avenida Professor Moraes Rego, Recife, State of Pernambuco 50670-901 Brazil

**Keywords:** Cardiotoxicity, ^18^F-FDG PET/CT, Anthracyclines, Nuclear medicine, Early diagnosis, Cardio-oncology

## Abstract

Despite advances in chemotherapy, the drugs used in cancer treatment remain rather harmful to the cardiovascular system, causing structural and functional cardiotoxic changes. Positron-emission tomography associated with computed tomography (PET/CT) has emerged like a promising technique in the early diagnosis of these adverse drug effects as the myocardial tissue uptake of fluorodeoxyglucose labeled with fluorine-18 (^18^F-FDG), a glucose analog, is increased after their use. Among these drugs, anthracyclines are the most frequently associated with cardiotoxicity because they promote heart damage through DNA breaks, and induction of an oxidative, proinflammatory, and toxic environment. This review aimed to present the scientific evidence available so far regarding the use of ^18^F-FDG PET/CT as an early biomarker of anthracycline-related cardiotoxicity. Thus, it discusses the physiological basis for its uptake, hypotheses to justify its increase in the myocardium affected by anthracyclines, importance of ^18^F-FDG PET/CT findings for cardio-oncology, and primary challenges of incorporating this technique in standard clinical oncology practice.

## Highlights

∙ CTX related to chemotherapy is an important cause of heart failure in surviving cancer patients;

∙ Cardiomyocytes may increase their glycolytic metabolism under significant stress. This phenomenon has been observed in both experimental and clinical studies;

∙ PET/CT is a powerful noninvasive diagnostic technique that provides both anatomical and metabolic information;

∙ ^18^F-FDG is a glucose analog that is the most commonly used radiopharmaceutical in oncological PET/CT exams;

∙ The degree of myocardial ^18^F-FDG uptake may be a promising marker of the cardiomyocyte changes that precede cardiac dysfunction in the CTX cascade.

## Introduction

Cancer and cardiovascular diseases are the most common causes of death in the world, regardless of social level [1]. Chemotherapy is a major contributor to the success of cancer therapy by reducing patient mortality and improving cancer prognosis [2]. However, the drugs used in cancer treatment can be detrimental to the overall health of patients, especially leading to cardiotoxicity (CTX) [3–8].

Anthracyclines (ATC), although are old drugs and rather harmful to the cardiovascular system, are effective in combating various types of cancers, and for this reason are still used widely [9]. The incidence rate of CTX associated with ATC varies from 5 to 48%, depending on the cumulative dose used [3]. Myocardial dysfunction with heart failure is the most alarming condition among this disease category, which has a significant morbidity and mortality [4, 10].

In terms of ventricular dysfunction, CTX is defined as a drop of more than 10% in the left ventricular ejection fraction (LVEF) in asymptomatic patients or more than 5% in symptomatic patients, with an absolute value below the standard reference of 50% [1, 5, 11]. However, this definition of CTX based on the degree of LVEF reduction ignores the changes that precede the LVEF reduction and all other toxic effects that occur in addition to this reduction. In 2016, the European Society of Cardiology revised the definition of CTX and extended it to any structural or functional changes in the heart and circulation, whether during the cancer treatment, immediately after it, or during the late posttreatment phase, chemotherapy, radiotherapy, or the disease itself as aggressive agents [1].

In addition to the initial cardiological evaluation with clinical history, physical examination, and electrocardiography, serum biomarkers evaluation and echocardiography are valuable for identifying cardiovascular complications associated with cancer treatment [5, 12]. However, all this approach presents limitations in documenting the initial cardiotoxic events and makes CTX an underdiagnosed clinical condition [5]. To predict that condition earlier, strategies using nuclear medicine molecular imaging have been explored [13, 14].

**Fig. 1:**
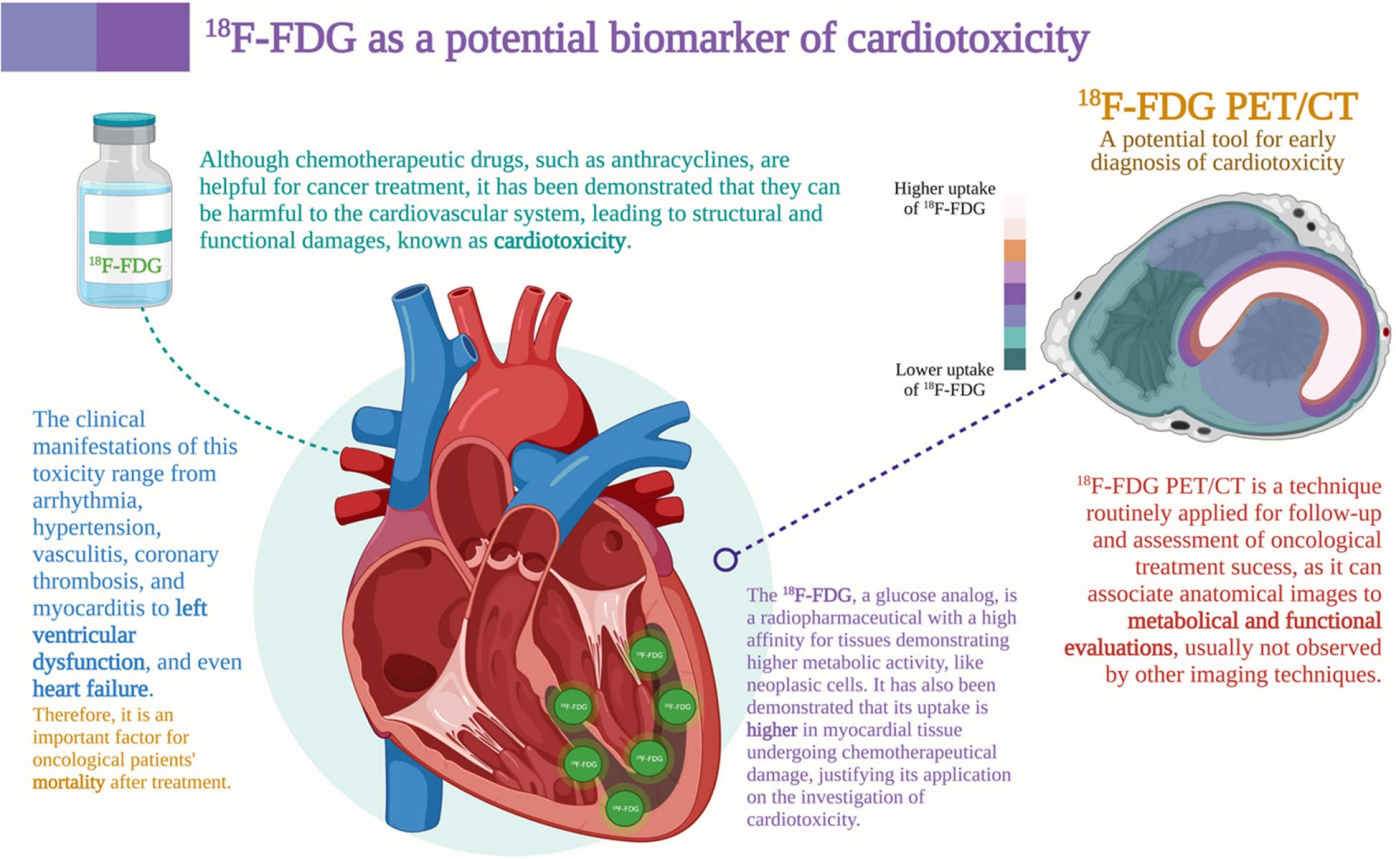
^18^F-FDG as a potential biomarker of cardiotoxicity. ^18^F-FDG: fluorodeoxyglucose labeled with fluorine-18; PET/CT: positron-emission tomography associated with computed tomography. Created with BioRender.com

Fluorodeoxyglucose labeled with fluorine-18 positron emission tomography associated to computed tomography (18F-FDG PET/CT) is a powerful nuclear medicine diagnostic technique that has already been routinely used in the staging, restaging, and therapeutic monitoring of various cancer types [15–19]. ^18^F-FDG is a glucose analog radiopharmaceutical that is transported into cells via glucose receptors. Previous studies have demonstrated that changes in the degree of ^18^F-FDG uptake by myocytes during or after the use of chemotherapeutic drugs reflect metabolic and mitochondrial changes that precede contractile dysfunction [15, 20, 21]. Thus, ^18^F-FDG PET/CT provides a unique access to myocardial metabolism and has emerged as a potential marker of CTX-related myocardial changes [22]. This review aimed to present the scientific evidence reported in the literature so far regarding the use of ^18^F-FDG PET/CT as an early metabolic marker of anthracycline related CTX (Fig. 1).

## Anthracycline cardiotoxicity

ATC are one of the classes of chemotherapeutic drugs that are most frequently associated with CTX. Members of this class include doxorubicin, daunorubicin, epirubicin, idarubicin, and mitoxantrone. They are used for the treatment of both hematological and solid malignant cancers, particularly breast cancer [23–25].

Although ATC have been recognized and used in chemotherapy for more than 60 years, to our knowledge, their action mechanisms underlying CTX development have not been clearly elucidated yet; in addition, whether the pathways for CTX development are the same as those used for tumor-cell damage remains unknown [24].

### Cardiotoxicity incidence

The incidence of this adverse effect, although relevant, is still very variable. One reason for this variability is dose-dependency [3]. Swain et al. demonstrated an incidence of CTX of 10%, 16%, 32% and 65% at cumulative doses of 250, 300, 400 and 550 mg/m2, respectively [26]. Age, sex and the presence of individual risk factors are also important in determining this possibility. Radiotherapy and/or concomitant treatments can lead to a higher incidence of CTX related to ATC in the population [27].

McGowan et al. reported an incidence of CTX of 2–4% of patients with congestive heart failure, 10% with structural heart changes, and 30–35% with cardiac biomarkers rise [28]. A retrospective study, analyzing three clinical trials with 640 patients, showed a 5% incidence of CTX [26]. A prospective study by Cardinale et al. with 2625 patients and a mean of 5.2 years of follow-up showed an overall incidence of 9% of patients affected by CTX after ATC therapy [29].

### Possible mechanisms (Fig. 2)

**Fig. 2:**
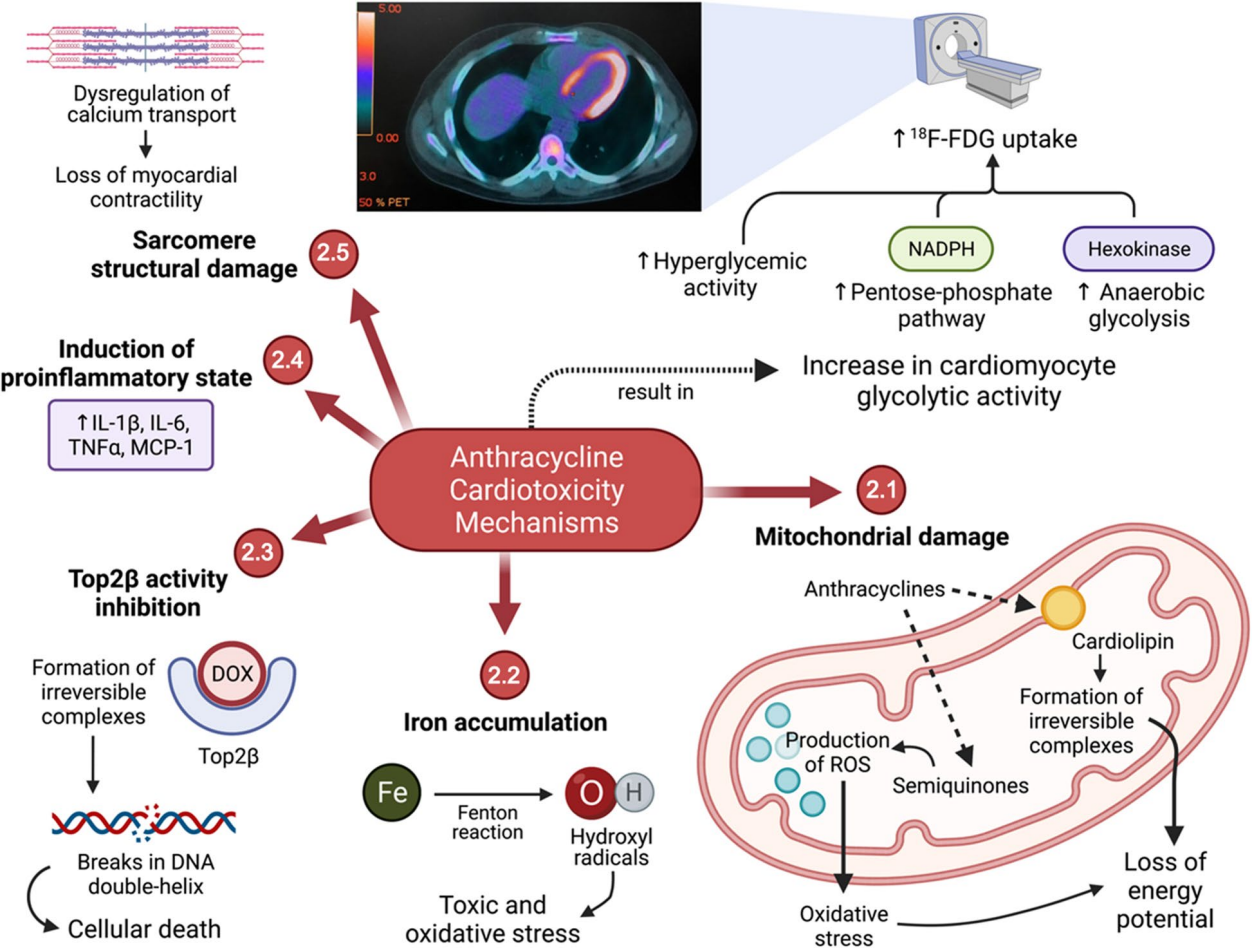
Suggested mechanisms of cardiotoxicity induction by anthracyclines and the effects on glycolytic metabolism in cardiomyocytes. ^18^F-FDG: fluorine-18-labeled fluorodeoxyglucose; DOX: doxorubicin; IL-1β: interleukin 1 beta; IL-6: interleukin 6; MCP-1: monocyte chemotactic protein 1; NADPH: reduced nicotinamide adenine dinucleotide phosphate; ROS: reactive oxygen species; TNFα: tumor necrosis factor alpha; Top2β: DNA topoisomerase 2-beta. In the upper portion of this figure, an axial slice of a ^18^F-FDG PET/CT examination performed after an anthracycline chemotherapy regimen can be observed (Source: personal collection). Created with BioRender.com

The most commonly accepted mechanism involved in CTX development is the generation of reactive oxygen species [23, 30]. The myocardium is already inherently and potentially vulnerable to oxidative stress because it has a lower basal level of antioxidant enzymes such as peroxidase, catalase, and superoxide dismutase in addition to containing 35–40% more mitochondria because of its high energy consumption [23, 30]. Moreover, the mitochondria ATC concentration can be up to twice as high as the extracellular medium, suggesting a certain avidity of this organelle for these drugs [31].

In this context of greater vulnerability, ATC quinones are then reduced to semiquinones through the action of mitochondrial enzymes such as NADH dehydrogenase [23, 31]. The electrons resulting from these reactions are then captured by oxidizing agents such as oxygen itself, forming superoxide radicals or even highly reactive hydroxyl radicals by contact with iron [23, 30, 31]. This theory of oxidative stress is supported by direct observational studies on doxorubicin-induced reactive oxygen species production measured using electron spin resonance spectroscopy as well as by an increase in oxidative stress markers in the cardiac tissue, including malonaldehyde—a product of lipid peroxidation [31].

Another hypothesis is that ATC promote iron accumulation in cardiomyocytes, leading to damage caused by their toxicity and to ferroptosis. This hypothesis was proposed after clinically verifying the cardioprotective effects of dexrazoxane, an iron chelating agent, when in association with chemotherapeutic agents [31, 32]. Although the mechanism for this accumulation remains ambiguous, it is believed to occur through the formation of a complex between iron and some drugs such as doxorubicin as well as through the downregulation of iron-exporting mitochondrial proteins such as mitochondrial ATP-binding cassete transporter 8 (ABC8) [23, 30].

However, recent evidence has presented a possibility that the cardioprotective effects of dexrazoxane are actually associated with the inhibition of complex formation between DNA topoisomerase 2-beta (Top2β) and ATC [32]. Top2β is an enzyme constitutionally present in quiescent cells such as cardiomyocytes. The presence of ATC leads to the formation of complexes with this enzyme, thus inhibiting its activity. Consequently, breaks occur in the double-helix structure of DNA, resulting in cell death by p53 activation [32, 33]. This mechanism of ATC-induced CTX is already well established and supported by the animal studies conducted by Zhang et al. [33], in which resistance to heart damage by these drugs was observed in rats with a specific deletion of Top2β.

Other mechanisms have also been proposed for anthracycline-induced CTX, such as the induction of a proinflammatory state, a dysregulation of calcium transport due to structural lesions of the sarcomeres, or a reduction in mitochondrial energy potential through the formation of irreversible complexes with cardiolipin—a phospholipid of the internal mitochondrial membrane that participates in the electron transport chain [23, 31, 32]. In general, structural damage to the energetic, metabolic, and genetic functions of cardiomyocytes is observed, which leads to an energy deficit and the activation of signals for apoptosis.

Therefore, a reduction in ^18^F-FDG uptake should be expected in the affected myocardium because most ATPs in its cells come from the electron carrier chain, which is compromised by continuous oxidative damage to the mitochondria. However, in animal and human studies, the opposite is actually observed: ^18^F-FDG uptake is higher in the myocardium of patients undergoing chemotherapy [5, 34–37].

Like the aforementioned mechanisms for CTX, those for the increased glycolytic metabolism in affected cardiomyocytes have not been well established yet and are mere hypotheses. One possibility is an increase in anaerobic glucose catabolism since the early stages in response to the loss of energy potential by cardiomyocytes. This increases the hexokinase activity, leading to greater ^18^F-FDG retention in the intracellular environment [35, 36]. Another recent hypothesis suggests that a higher myocyte glucose intake is associated with an acceleration of the pentose phosphate pathway—an alternative route in the cytosol that produces energy in the form of reduced NADPH—and is a mechanism of opposition and protection against injuries due to excess reactive oxygen species [34, 35].

Although the impact of ATC on cardiac glycolytic metabolism remains poorly clarified, it is increasingly observed in more recent evidence. Sabatino et al. [38] noted a significant CTX reduction in nondiabetic rats receiving doxorubicin associated with empagliflozin, a sodium-glucose co-transporter 2 (SGLT2) inhibitor, compared with a control group that did not receive this hypoglycemic agent. They observed lower reductions in LVEF and in myocardial fibrosis in the empagliflozin group, regardless of changes in glycemia. Thus, they proposed the hypothesis that SGLT2 inhibition is associated with significant changes in cardiomyocyte sodium homeostasis, which leads to the protection of the mitochondrial system and a better remediation of the oxidative stress induced by ATC [38].

Peng et al. [39] also found similar results for this association of ATC use with glycolytic metabolism when analyzing in vitro rat cardiomyocytes exposed to doxorubicin with or without the use of teneligliptin, a dipeptidyl peptidase 4 (DPP4) inhibitor. They observed that the use of doxorubicin was associated with a dose-dependent increase in DPP4 expression in these cells, which could be associated with an increase in the expression of proinflammatory cytokines. This increase was demonstrated as up to 4.5-fold increase in the concentration of interleukin 1-beta and up to 6 times increase in the concentration of monocyte chemotactic protein 1, resulting in up to 40% cell loss in the study samples [39]. When accompanied by teneligliptin administration, the therapy regimen induced only 20% loss in cell viability, associated with lower elevations in proinflammatory cytokines: an increase in interleukin 1-beta concentration by only 1.5–2.5 times and in monocyte chemotactic protein 1 concentration by 2–3 times compared with cells without doxorubicin use [39].

In other words, although there is no further clarification regarding the pathways of the alteration of glycolytic metabolism induced by ATC, there is probably an increase in glucose catabolism, represented by a stimulus to alternative glucose consumption pathways and by a greater expression of hyperglycemic molecules such as SGLT2 and DPP4 in cardiomyocytes. This metabolic increase can be observed on PET/CT with the administration of ^18^F-FDG, which acts as a glucose analog, as noted earlier.

### Diagnosis of CTX in clinical practice

#### Assessment of myocardial function and performance

##### Echocardiography 2D and 3D

**Fig. 3:**
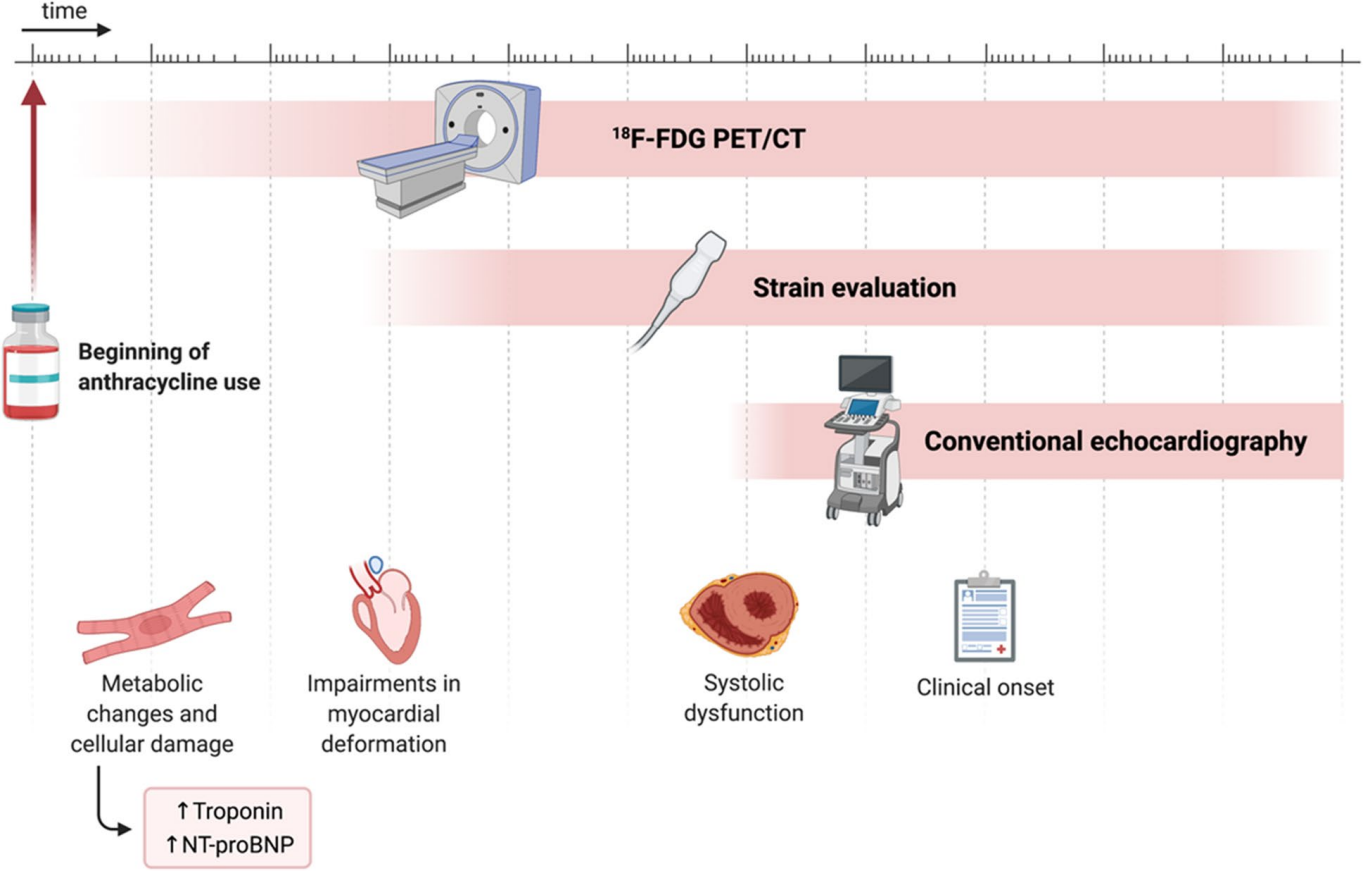
Evolution of anthracycline-induced cardiotoxicity and image tracking. ^18^F-FDG: fluorodeoxyglucose labeled with fluorine-18; NT-proBNP: N-terminal pro b-type natriuretic peptide; PET/CT: positron-emission tomography associated with computed tomography. Created with BioRender.com

Currently, the follow-up of patients undergoing chemotherapy with ATC and the diagnosis of CTX is made with clinical evaluation and echocardiography [1–5]. The measurement of LVEF is the most accepted for the diagnosis of systolic dysfunction related to cancer treatment [1, 3]. The assessment of ventricular function by imaging methods before the start of chemotherapy helps in the diagnosis of CTX and in the identification of patients at higher risk of cardiological complications during cancer treatment [40]. Usually, this assessment is performed through transthoracic echocardiography due to its low cost and easy accessibility [1, 11, 22]. Some studies shows that 3D-echocardiography provides more accurate measurements of LVEF and left ventricular (LV) volumes than 2D-echocardiography [41]. However, the identification of CTX, based only on the decrease in LVEF, occurs late, since ventricular dysfunction with an impact on LVEF only occurs after significant injury to the myocardial tissue. Thus, LVEF measurement has been considered a low sensitive marker of CTX [11, 22, 40]. For this reason, earlier ways to monitor and stratify these patients have been proposed, as shown in Fig. 3.

In addition, the sensitivity of traditional echocardiography to detect longitudinal variations in systolic function is still low, mainly due to frequent variations in pre- and afterload during chemotherapy and the intra- and interobserver variability that can reach up to 10% (precisely one of the parameters accepted for the diagnosis of CTX) [41]. Echo3D has strong agreement with cardiac magnetic resonance (CMR), but has reduced availability, high cost and need for trained professionals [1, 11].

##### Global Longitudinal Strain (GLS)

Another echocardiographic measure that has been extensively studied in recent years is the degree of myocardial deformation (strain) by Speckle Tracking [42–44]. Among the parameters of myocardial deformity analysis, the one that has been shown to be of better clinical utility is the global longitudinal strain (GLS) that precede LVEF decline and would be predictive of subsequent CTX and cardiovascular events in asymptomatic and symptomatic LV dysfunction patients [42, 45]. GLS is useful in detection of subclinical LV dysfunction during therapy [22]. A reduction of more than 15% in GLS, immediately after or during treatment with ATC, is the most useful parameter in predicting CTX, while a reduction of less than 8% would likely exclude the diagnosis of CTX [45].

A recent meta-analysis evaluating the prognostic value of GLS for early prediction of chemotherapy-induced CTX supports the American Society of Echocardiography and European Association for Cardiovascular Imaging recommendation for the routine use of GLS in the monitoring of patients undergoing therapy for cancer treatment [46]. However, the limitation of acoustic windows, heart rate and rhythm, concentric ventricular hypertrophy, pacemaker rhythm, at least moderate aortic and mitral valve diseases may difficult the measurements [22, 43–45, 47]. As the calculation of GLS varies between vendors of Echo machines and analytical equipment and software, it is recommended to use the same system to be able to accurately compare values over time [22].

##### Equilibrium radionuclide angiocardiography (ERNA)

Another methodology of evaluating LVEF include ERNA or multigated acquisition scan (MUGA) that has low inter and intra observer variability, in addition to the excellent accuracy and serial reproducibility, and is not limited by body habitus, acoustic windows, or implantable devices [15, 48]. ERNA has the disadvantage of radiation exposure, low availability, and other cardiac structures such as valve and pericardium are not evaluated [1, 11, 49].

##### Cardiac magnetic resonance (CMR)

The gold standard technique for quantification of LVEF is CMR with more accurate measurements of LVEF and LV volumes than echocardiography. T1 mapping and extra cellular volume, T2 mapping (with increased relaxation time) and myocardial strain can identify CTX patients prior to LVEF decline and are now part of armamentarium in cardiovascular follow-up [22]. CMR imaging is useful in patients with limited acoustic window and borderline LVEF. However, the CMR is limited by low availability and high costs, in addition to difficulties to perform the exam, such as claustrophobia and artifacts due to high heart rate or arrhythmias and the presence of implantable devices [11, 12, 47].

#### Cell damage assessment

All previously described methods detect myocardial dysfunction. The chase to identify minimal cell damage and high-risk patients to develop CTX has been proposed in several studies [13, 50, 51]. Guidelines already recommended the measure of high-sensitivity cardiac troponins and natriuretic peptides for high-risk patients or undergoing potentially cardiotoxic therapy [1–3]. The increase in troponin I after chemotherapy treatment has been shown to be a risk marker for cardiac events [50, 51]; isolated elevations without imaging parameters may be considered as biochemical evidence of CTX [52] and help to identify patients who may benefit from use of angiotensin-converting enzyme inhibitors (ACE-Is) [53].

A meta-analysis of 61 trials including 5691 patients assessed the utility of cardiac troponins or plasma brain natriuretic peptide (BNP) and N-terminal proBNP (NT-proBNP) levels in adult patients undergoing cancer therapy. This study showed that elevated troponin levels predict LV dysfunction, but failed to show association with BNP/NT-proBNP levels [54].

However, measurement of natriuretic peptides can help establish or exclude the diagnosis of symptomatic heart failure in the acute and subacute setting [52]. Moreover, persistent elevation of BNP may be an indication of adverse cardiac outcomes [55, 56]. Clinical studies have used the measurement of plasma BNP and NT-proBNP as biomarkers of CTX. Mean BNP/NT-proBNP levels were increased in patients post-treatment, but the available evidence did not consistently indicate prediction of LV dysfunction [54].

Cardioprotection strategies can be guided by changes in these complementary exams. Despite this wide applicability, the variety of troponin and NT-pro-BNP assays should pay particular attention to the management of these patients. Furthermore, studies are still insufficient to establish the meaning of sudden elevations of these markers. Their role in routine monitoring and surveillance is not yet clearly established [13].

## Role of ^18^F-FDG PET/CT in the diagnosis of CTX by ATC

Some recent studies have highlighted the potential role of ^18^F-FDG PET/CT in the early detection of myocardial injury by chemotherapy and radiotherapy [15, 21, 35, 36]. Assuming that myocardial injury by oncological treatment induces an increase in glycolytic metabolism, this metabolic alteration can be detected by measuring cardiac ^18^F-FDG uptake and, therefore, be considered an early marker of CTX [57]. Because metabolic changes precede anatomical changes in diseases, CTX-associated metabolic changes in cardiomyocytes can be quickly observed by analyzing the pattern of ^18^F-FDG consumption in ^18^F-FDG PET/CT examinations.

### Physiological basis

^18^F-FDG PET/CT is a noninvasive diagnostic technique that provides both anatomical and metabolic information [17, 58, 59]. Briefly, in this examination, the equipment three-dimensionally captures the radiation emitted by the radiopharmaceutical administered into the patient. This allows the observation and quantification of metabolic activity in target organs and lesions. Morphological analysis are simultaneously performed through the emission and detection of X-rays by CT coupled to PET [17]. Thus, the images obtained through PET are based on the in-vivo biodistribution of the radiotracer together with the simultaneous anatomical CT images [60]. The use of magnetic resonance imaging (MR) in combination with PET (PET/MR) can also significantly contribute to anatomical and functional assessments; however, this type of equipment is still extremely expensive and scarcely available [61].

The first studies regarding ^18^F-FDG were conducted in the 1970s [62, 63]. The initial models already revealed the ability of studying glucose metabolism in the myocardium. In 1976, a distribution model of ^18^F-FDG was developed [62]. Since then, the concentration of ^18^F-FDG in the myocardial tissue has been observed to rise within the first minute of ^18^F-FDG administration [63]. The same distribution was observed in previous studies [62, 64]. Clinical studies progressed and a more assertive determination of the dynamics and behavior of ^18^F-FDG in cell chemistry became possible [63].

After being injected into the body, ^18^F-FDG is taken up by cells through the membrane glucose transporter (GLUT) [59, 65]. GLUTs are probably the best studied human transporter proteins because of their important role in glucose uptake [66]. In the healthy hearts, GLUT-4 is present in the cardiomyocyte membrane and is primarily responsible for the inflow of glucose, mainly in situations of increased plasma insulin concentration [67]. In clinical context of tissue inflammation, such as myocarditis, and sarcoidosis, GLUT-4 is suppressed by preparing the patient through prolonged fasting and a high-fat, low-carbohydrate diet approximately 24 h before the examination, whereas GLUT-1 and GLUT-3 are overexpressed in immune system cells and are not influenced by fasting and diet, thereby increasing the influx of glucose to the myocardium [68, 69]. Regarding neoplasm behaviors, GLUT-1 plays a fundamental role in mediating the uptake of ^18^F-FDG by tumors [70]. The excessive expression of this carrier has been demonstrated in various cancer types (e.g., lymphoma, breast cancer, lung cancer, pancreatic cancer, and others) [71, 72].

After being incorporated into the intracellular environment by the GLUTs, ^18^F-FDG undergoes phosphorylation by the action of hexokinase, resulting in the formation of fluorodeoxyglucose (FDG)-6-phosphate. Therefore, ^18^F-FDG concentration increases with the increase in glycolysis rate in tissues (as a consequence of GLUT expression) [59, 65, 73]. This increased concentration should be detected using PET; in combination with the anatomical evaluation provided by CT, ^18^F-FDG PET/CT examination provides accurate information regarding the anatomical site with increased glycolysis.

### Scientific evidence (studies)

^18^F-FDG PET/CT has been widely used in oncology. The purpose of the exam is to evaluate the tumor uptake of the ^18^F-FDG radiotracer, which reflects the degree of cellular metabolism [16–19]. This technique is indicated for the detection, staging, follow-up and monitoring of the treatment response in patients with cancer [16, 74, 75]. In addition, cells under significant stress may increase their glycolytic metabolism; this phenomenon has been observed in heart cells in both experimental and clinical studies [21, 34–36, 76, 77].

A few of these retrospective studies [21, 34, 77] have demonstrated the possible contribution of ^18^F-FDG toward CTX assessment, as summarized in Table 1. Borde et al. [21] analyzed the myocardial uptake of ^18^F-FDG in patients with lymphoma who were treated with doxorubicin. They reported that a significant increase in the myocardial uptake of ^18^F-FDG was an early marker of CTX in patients who received doses higher than 250 mg/m^2^. Bauckneht et al. [34] assessed 69 patients with Hodgkin's lymphoma and demonstrated a progressive increase in the cardiac uptake of ^18^F-FDG during treatment. This increase persisted in the 6-month follow-up after the completion of chemotherapy. Interestingly, they hypothesized that a low myocardial uptake of ^18^F-FDG before treatment with doxorubicin predicts CTX development.

**Table 1:** Summary of the leading studies using ^18^F-FDG PET/CT in the detection of cardiotoxicity

Author/Year	Objectives	Sample	Key Results	Conclusion
Borde et al., 2012 [21]	To evaluate changes in myocardial FDG uptake in patients undergoing DOX therapy and investigate the clinical significance of these changes	18 patients with lymphoma who were treated with DOX	12 patients presented significant changes in the mean myocardial SUV compared with baseline PET/CT	Presence of three patterns of postchemotherapy myocardial uptake alteration with DOX
Bauckneht et al., 2017 [34]	To assess whether follow-up with serial PET/CT examinations can predict CTX from DOX	69 patients with non-Hodgkin lymphoma undergoing ABVD therapy	Progressive increase in LV SUV throughout DOX therapy. Cardiac changes appeared in 31% of patients, and the baseline SUV was lower in these patients	LV SUV rises as the treatment with DOX progresses, and this increase has a dose-dependency ratio. This elevation is also greater in patients showing a low uptake on baseline PET/CT
Sarocchi et al., 2018 [36]	To evaluate changes in myocardial FDG uptake in patients undergoing DOX therapy and compare these changes with a posttreatment echocardiogram	43 post-chemotherapy patients who received the first-line treatment including doxorubicin	Gradual increase in LV SUV before, during, and after chemotherapy. Post-chemotherapy echocardiographic LVEF was lower in patients with lower myocardial FDG uptake	Chemotherapy with DOX leads to increased myocardial ^18^F-FDG uptake, and this increase is associated with a reduction in LVEF
Kim et al., 2020 [76]	To evaluate changes in myocardial ^18^F-FDG uptake and establish a relationship between these changes and CTX secondary to anthracycline and trastuzumab uses	121 patients treated with anthracyclines or trastuzumab	15 patients with CTX, in whom more diffuse LV uptake, higher overall SUV, and RV SUV were observed	RV FDG uptake and RV SUV were associated with CTX
Takanami et al., 2020 [78]	To identify the clinical implications of myocardial ^18^F-FDG uptake patterns	86 ^18^F-FDG PET/CT examinations associated with myocardial perfusion tests	89% of the examinations with an elevated focal pattern and 89% of those with focal defects had abnormal myocardial perfusion	^18^F-FDG uptake with an elevated focal pattern or a focal defect in the myocardium correlated with abnormal myocardial perfusion
Haider et al., 2020 [79]	To assess whether the myocardial ^18^F-FDG uptake pattern can be used to estimate CTX risk	302 patients receiving cancer treatment	Focal myocardial ^18^F-FDG uptake was a strong predictor of ongoing ischemia and myocardial scarring	A focal uptake pattern indicates a significantly increased risk for multiple myocardial abnormalities
Dourado et al., 2021 [77]	To investigate chemotheraphy effects on heart by using ^18^F-FDG uptake before, during and/or after chemoteraphy	70 patients with lymphoma	A percentage increase ≥ 30% of LV SUVmax occurred in more than half of the sample	A clear increase in cardiac ^18^F-FDG uptake in patients with lymphoma, during and/or after chemotherapy

^*18*^*F-FDG* Fluorine-18-labeled fluorodeoxyglucose, *ABVD* Association of Adriamycin® (doxorubicin), bleomycin, vinblastine, and dacarbazine, *CTX* Cardiotoxicity, *DOX* Doxorubicin, *LV* Left ventricle, *LVEF* Left ventricular ejection fraction, *PET/CT* Positron-emission tomography associated with computed tomography, *RV* Right ventricle, *SUV* Standard uptake value, *SUVmax* Maximum standard uptake value

In 2018, a study assessing patients with Hodgkin's lymphoma and a primary chemotherapy regimen containing doxorubicin showed that the LV standard uptake value (SUV) of ^18^F-FDG gradually increased from the basal PET compared with the interim PET and when comparing the latter to the post chemotherapy PET [36]. In the subgroup of patients who had undergone both pre- and post-chemotherapy echocardiography, no significant difference was noted in the mean LVEF and LV end-diastolic diameter between those two evaluations. However, when patients were categorized as having higher or lower LV SUV than the mean LV SUV, the LVEF was significantly lower in those with high LV SUV. Likewise, the LVEF was also significantly lower on post-chemotherapy echocardiography in patients showing high LV SUV on the interim or final ^18^F-FDG PET/CT [36].

A recent evaluation of 121 patients with breast cancer who underwent oncological ^18^F-FDG PET/CT and echocardiography both before and at the end of chemotherapy showed that the group that developed CTX tended to show an increased LV uptake of ^18^F-FDG, with a diffuse pattern of hyper uptake. The uptake remained high for at least one year [76]. This recent study also showed a significant association between ^18^F-FDG uptake in the right ventricle wall and CTX development [76]. A right ventricle wall SUV of > 1.8 and a variation in the right ventricle wall SUV of at least 0.4 during chemotherapy were associated with CTX.

Takanami et al. [78] also evaluated myocardial ^18^F-FDG uptake patterns in oncological ^18^F-FDG PET/CT, demonstrating that the pattern of focal myocardial hyper uptake or focal defect in the diffuse myocardial hyper uptake of ^18^F-FDG correlates with subsequent abnormal findings on myocardial perfusion scintigraphy. Another retrospective study with 302 patients who underwent oncological ^18^F-FDG PET/CT and subsequent myocardial perfusion scintigraphy revealed that the pattern of focal myocardial uptake of ^18^F-FDG in oncological ^18^F-FDG PET/CT examinations can predict patients at high risk of cardiovascular complications who would benefit from a more careful cardiological follow-up [79].

Dourado et al. [77] retrospectively evaluated 70 patients with lymphoma submitted to ^18^F-FDG PET/CT. They observed an increase in LV maximum SUV (SUVmax) from 3,5 ± 1,9 (baseline) to 5,6 ± 4,0 (interim), *p* = 0.01, and from 4,0 ± 2,2 (baseline) to 6,1 ± 4,2 (post-therapy), *p* < 0,001. A percentage increase ≥ 30% of LV SUVmax occurred in more than half of the sample [77]. The rise of cardiac SUV was accompanied by an increase in LV SUVmax/Aorta SUVmax and LV SUVmax/Liver SUVmean (mean standard uptake value) ratios. These results show a clear increase in cardiac ^18^F-FDG uptake in patients with lymphoma during and/or after chemotherapy.

## Advantages, limitations, and future directions

The applications of ^18^F-FDG PET/CT can reach beyond the mere identification and monitoring of CTX, providing an outline of the mechanisms of CTX pathophysiology. The ability of ^18^F-FDG to selectively track the pentose phosphate pathway response to oxidative stress in the endoplasmic reticulum can indicate metabolic abnormalities, identifying early continuous myocardial damage and avoiding the distressing contractile dysfunction [80].

Recognizing a possible underlying disease based on the knowledge of common ^18^F-FDG myocardial uptake patterns can help improve patient management by avoiding cardiac complications. An important advantage of using this biomarker is that it is already routinely used in oncologic patients to scan cancer in the patient's body and monitor treatment responses through the degree of ^18^F-FDG uptake by the tumor and/or metastasis [16–19]. The information about CTX can be get from an already schedule ^18^F-FDG PET/CT scan, without exposing the patient to additional radiation.

Because this examination is performed serially in patients with cancer to monitor the neoplasm, guidelines for preparation for the examination should be the same at different evaluation times. Therefore, patients should be considered their own controls. This increases the accuracy of their examination results, including the myocardial ^18^F-FDG uptake pattern before and after the treatment.

However, some challenges need to be overcome before implementing this test in cardio-oncological clinical practice.

A recent review of the changes in myocardial uptake of ^18^F-FDG in PET/CT performed without preparation to suppress the physiological uptake of ^18^F-FDG by the myocardium stated that various physiological uptake patterns may be present [81]. Factors such as sex, age, obesity, the presence of diabetes mellitus, and the use of medications (i.e., bezafibrate, levothyroxine, thiazolidinedione, and benzodiazepine) can also influence cardiac ^18^F-FDG uptake; these factors should be considered for the correct interpretation of the changes found in routine examinations. The cardiac ^18^F-FDG uptake is not specific for CTX, and its increase may be related to inflammatory disease or others cardiomyopathies, which may be a transient adaptation and not translate to permanent cell injury [81].

Whether there is a cutoff point for an increase in cardiac ^18^F-FDG SUV after chemotherapy that indicates clinically relevant CTX remains unknown. Another point that should be clarified is the best SUV assessment site in the heart walls. Should this be the free right ventricular wall, interventricular septum, or lateral LV wall, for example, or should the entire heart area be analyzed? Several pathological conditions are known to increase glucose intake by the cardiac muscle, including ischemia and inflammation [65, 73]. Preparation with low-carbohydrate, high-fat diet 24 h before the exam also helps to reduce the physiological myocardial ^18^F-FDG uptake [49]. Other possibilities that should be discussed are whether this phenomenon of increased myocardial ^18^F-FDG uptake is transient and whether it is only a cardioprotective mechanism, which would be reversed after cell damage is resolved. In addition, is there a direct relationship between increased myocardial ^18^F-FDG uptake and sustained myocardial loss? If so, what is the nature of this relationship and how soon can this myocardial loss be estimated?

## Conclusion

The delay in the diagnosis of CTX after chemotherapy when using only conventional imaging techniques, in addition of the absence of therapeutic measures during this period may result in a loss of myocardial tissue and, consequently, the worsening of disease prognosis in these patients, despite the oncological improvement. To mitigate this delay in diagnosis and initiate therapeutic measures earlier, metabolic assessment appears to be a promising technique, however more studies on ^18^F-FDG PET/CT should be performed to better clarify its role in this context.

## Data Availability

Not applicable.

## Abbreviations

^18^F-FDG: Fluorodeoxyglucose labeled with fluorine-18
ACE-Is: Angiotensin-converting enzyme inhibitors
ATC: Anthracyclines
BNP: Brain natriuretic peptide
CMR: Cardiac magnetic resonance
CTX: Cardiotoxicity
DNA: Deoxyribonucleic acid
DPP4: Dipeptidyl peptidase 4
ERNA: Equilibrium radionuclide angiocardiography
GLS: Global longitudinal strain
GLUT: Glucose transporter protein
HER2: Human epidermal growth factor receptor type 2
LV: Left ventricular
LVEF: Left ventricular ejection fraction
MUGA: Multigated acquisition scan
NADH: Reduced form of nicotinamide adenine dinucleotide
NADPH: Reduced form of nicotinamide adenine dinucleotide phosphatase
NT-proBNP: N-terminal brain natriuretic peptide
p53: Phosphoprotein 53
PET/CT: Positron-emission tomography associated with computed tomography
SGLT2: Sodium-glucose co-transporter 2
SUV: Standard uptake value
*Top2β*: Topoisomerase 2-beta
